# Huge Task for Ministry of Pensions

**Published:** 1918-12-14

**Authors:** 


### DEMOBILISING MILITARY HOSPITALS.

### Huge Task for Ministry of Pensions.

An announcement was made on the 10th instant by the Secretary of the War Office that, under instructions from the War Cabinet, it has been decided that, for a period of six months from December 3, 1918, the War Office will continue to afford treatment in military hospitals for all deserving soldiers requiring prolonged treatment, and will also provide accommodation and treatment for pensioners during that period. The medical correspondent of *The Times*, in referring to this announcement, was apparently under the impression that the course has to some extent been taken in consequence of representations made in our powerful contemporary, but it has been an open secret for several months that the military hospitals would continue as military hospitals for six months after the cessation of hostilities, and that the patients in them would then be handed over to the care of the Ministry of Pensions.

The task that the Ministry has before them is not merely a question of taking over many hospitals and a vast number of patients, but one of changing the character of the system under which those hospitals are managed. As *The Times* correspondent points out, the patients will in every sense become civilians, and discipline, as understood in military establishments, will not, therefore, obtain. Of course the Navy and Army will still have in their charge a number of hospitals. This was the case before the war, and, in view of the probability of a much larger standing Army and an undiminished Navy, the normal hospital accommodation required may, if anything, be larger than in pre-war days. The task, however, of the Ministry of Pensions will not be affected by this fact.

There are no recent figures to show how many beds are occupied by sailors and soldiers in hospitals at the present moment, but in one command alone, the Eastern Command, there are between 80,000 and 90,000 patients. During the six months before us there will be an opportunity to dispose of the slightly wounded men by discharging them to their own homes, and of the permanently helpless patients, such as paraplegics, by placing them in homes and similar institutions. The residue of patients will be sick and wounded whose hospital treatment started before the close of hostilities, and should be in most cases well on the way to a conclusion.

But, in addition, provision must be made for cases of accident and illness arising during demobilisation which, in respect of several million men, must be fairly numerous. These cases will resemble the ordinary casualties of hospital practice; at the end of six months the bulk of the patients are not likely to differ from the generality of cases in voluntary hospitals, and the proportion of "heavy cases" will probably be small. It is quite reasonable to expect that the work of the Ministry of Pensions, owing to these facts, as well as to the demobilisation of Army doctors, nurses, orderlies, and other military *personnel*, will be carried on, in respect to organisation, on voluntary hospital lines.

Many of the buildings now used as military hospitals have been borrowed from local authorities and, as soon as possible, will have to be returned; others are private houses, and again many patients are being cared for in the voluntary hospitals where, in most instances, beds previously allocated to civilian patients have been temporarily diverted from usual hospital purposes. The task of the Ministry of Pensions is not confined entirely to taking over the patients, but to providing or re-arranging accommodation for them. For economical reasons a great majority of the small hospitals managed by the Red Cross will doubtless be closed, but in view of the question of providing for satisfactory working the size of the hospitals chosen may be moderate rather than large.

In looking through particulars of the 527 hospitals in the Eastern Command we notice that 404 of them are hospitals of under 100 beds, the average size being forty-two. As every possible building has been considered with a view to its use for hospital purposes, these figures, which would no doubt be approximately the same in other Commands, may be accepted as illustrative of the general accommodation for sailor and soldier patients.

The medical correspondent of *The Times* points out that it is essential to make use of the special experience of Army doctors, especially in view of the return to civil life of large numbers of men with war infections which it has been impossible to eradicate. These are often patients who are likely to suffer from further attacks of their disease or from the chronic ill-health which must inevitably follow it. Amongst these may be mentioned cases of malaria and dysentery. These men plainly cannot remain in the Army, but in civil life they are unlikely to find the special knowledge and care which they require. This is clearly a point where specialism is required, and where it no doubt will be forthcoming.

If the general practitioner is allowed to return to his long-suffering practice, and the young medical man is permitted to pick up the threads of his interrupted education, the Ministry may have difficulty in finding a sufficiency of medical officers. The nursing profession as a whole is tired out, but the trained nurse, no doubt, acting up to her traditional courage, will persevere to the end. Even the V.A.D. worker, who in the past has proved so useful, is showing signs of losing zest. Altogether the task of securing sufficient and efficient staffs for the new hospitals will be no light one. But difficulties far greater have been overcome during the past few years and, the general principles under which the new scheme is to be worked having been decided upon, we have no doubt that our disabled men, when discharged, will receive the care and skilled attention that their cases need, and that their countrymen are determined to provide.

